# Coordinated Target Tracking via a Hybrid Optimization Approach

**DOI:** 10.3390/s17030472

**Published:** 2017-02-27

**Authors:** Yin Wang, Yan Cao

**Affiliations:** 1 State Key Laboratory of Virtual Reality Technology and Systems, Beihang University, Beijing 100191, China; 2 College of Astronautics, Nanjing University of Aeronautics and Astronautics, Nanjing 210016, China; caoyan626@126.com

**Keywords:** unmanned aerial vehicles, UAV cooperation, persistent tracking, evolutionary algorithm

## Abstract

Recent advances in computer science and electronics have greatly expanded the capabilities of unmanned aerial vehicles (UAV) in both defense and civil applications, such as moving ground object tracking. Due to the uncertainties of the application environments and objects’ motion, it is difficult to maintain the tracked object always within the sensor coverage area by using a single UAV. Hence, it is necessary to deploy a group of UAVs to improve the robustness of the tracking. This paper investigates the problem of tracking ground moving objects with a group of UAVs using gimbaled sensors under flight dynamic and collision-free constraints. The optimal cooperative tracking path planning problem is solved using an evolutionary optimization technique based on the framework of chemical reaction optimization (CRO). The efficiency of the proposed method was demonstrated through a series of comparative simulations. The results show that the cooperative tracking paths determined by the newly developed method allows for longer sensor coverage time under flight dynamic restrictions and safety conditions.

## 1. Introduction

Recently, unmanned aerial vehicles (UAV) have been widely used in both defense and civilian applications. In many of these applications, the UAV is required to continuously track a moving target, such as in the surveillance and tracking of ground objects. This task becomes challenging when the target is moving in a complex and dynamic environment, where the target may be occluded by ground features, such as buildings and mountains. In the case where the tracking cannot be achieved by using a single UAV, multiple UAVs may be deployed to improve the tracking robustness. The methods to coordinate the movements of multiple UAVs can be generally grouped into two categories, namely centralized and distributed algorithms, respectively [1]. In centralized methods, the mission of each UAV is dispatched from a central station or agent based on global information collected from the all of the aircraft. These methods generally tend to produce better solutions since the global information is available. In contrast, the distributed approaches determine the motion of each UAV by using the local information obtained from the UAV itself and its neighboring UAVs, which enjoys the advantage of computational efficiency and robustness when compared with their centralized counterparts. In a moving target tracking problem, besides generating the tracking path for each UAV, the motion of the target should be estimated since such information cannot be obtained from prior knowledge. As the purpose of this paper is to determine the optimal path of each UAV for cooperative tracking tasks, the motion pattern of the target is assumed to be available when it can be observed by the tracking UAVs. 

The aim of cooperative tracking is to keep the target under the coverage of the UAV sensors at all time. To address this problem, Yang et al. adopted the Lyapunov Vector Field Control (LVC) approach to make the unmanned aerial vehicles converge to a circular area on top of the target [2]. However, this method does not take environmental factors into account, such as non-fly zones and the occlusion of sensors’ field of view. Besides, in their method prior knowledge about the motion of the target is required, which is not available in most cases. In order to cope with this limitation, Belkhouche et al. [3] proposed to apply active avoidance strategies for obstacles in the environment, and determine feasible fly-zones by calculating the speed and position of the UAV relative to the obstacle. Cheng et al. presented a time-related optimal path planning algorithm to determine the cooperative tracking trajectories for each UAV [4]. Quintero and colleagues adopted a dynamic programming technique to minimize the distance error covariance for solving the cooperative tracking task, however the relative angle between UAVs and the target was ignored [5]. Chen et al., presented a hierarchical method, combining the Lyapunov Guidance Vector Field (LGVF) and Tangent Guidance Vector Field (TGVF) approaches, to solve the path planning problem for coordinated tracking [6]. In this method, the TGVF algorithm is firstly utilized to determine an optimal path from the initial point to a limiting circle, and then UAV would converge to the desired limit circle by optimizing the heading command through the use of the LGVF approach. 

As discussed previously, due to the complexity and uncertainty of the ground environment, the target may be out of the sight of the UAV sensors, thus the motion of the target should be estimated. Shaferman et al. considered the sensor field-of-view (FOV) convergence in the urban environment, and determined an optimal path by maximizing the sensor convergence time to the target [7]. In order to improve the tracking performance of unmanned aerial vehicles (UAVs) under unknown conditions. Yu and his colleagues proposed an online path planning algorithm by estimating the location of the target based on its past states using a Bayesian filter based method [8]. Zhang et al., applied the Model Predictive Control (MPC) concept to solve the cooperative path planning problem with unknown target motions [9]. Beard and his co-workers present a decentralized cooperative aerial surveillance method by solving the associated combinational optimization problem, where both flight dynamics and environmental constraints are taken into account [10]. He and Xu proposed a model predictive control-based solution for the cooperative tracking path planning problem by considering urban area occlusions, however, the secure distances between UAVs were not taken into account [11]. 

Evolutionary algorithms, such as genetic algorithm approaches, also applied to solve the cooperative path planning problem [12,13,14], achieve good results in many offline path planning applications. Wise et al., compared commonly used methods for cooperative tracking path planning problems and in their work [15], readers can find a comprehensive review of the relevant literature.

This work present a novel cooperative tracking path planning approach for the visual tracking tasks of multiple UAVs, by coupling the receding horizon control principle and chemical reaction optimization framework [16]. The proposed method does not require any prior information about the motion and trajectory of the targets, and is capable of dealing with the occlusion of the sensors’ field of view. Followed by this Introduction, the kinematics model of UAV and sensor visible regions model are described in Section 2. Section 3 presents the proposed method in great detail, and we demonstrate the performance of the proposed method through a series of comparative simulations in the following section. Finally, we conclude this work and discuss possible future work directions in Section 5.

## 2. Problem Description 

### 2.1. Kinematics Model of UAV

**Figure 1:**
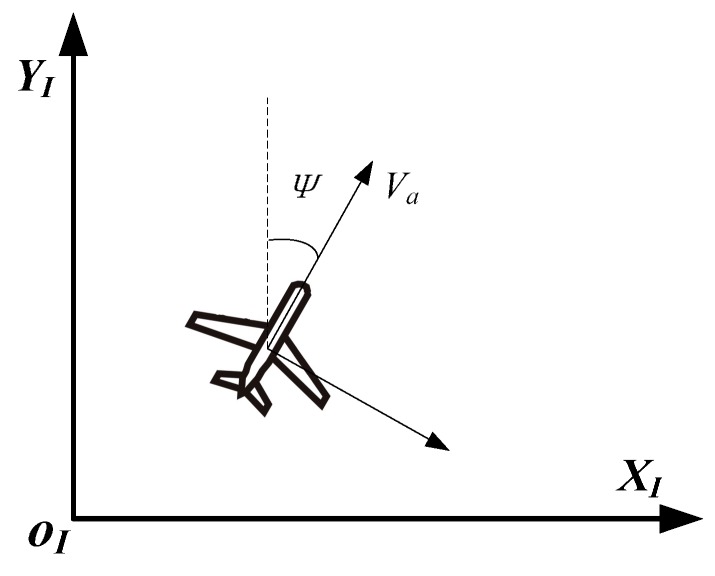
Schematic diagram illustrates the coordinate of the Dubins model.

Assuming the UAVs are flying at a constant altitude with a fixed speed, the motion of the UAV can be reduced to a 2-dimensional Dubins model. Figure 1 shows the 2D planar view of the Dubins coordinate system, where *X_I_*, *Y*_I_ are the Cartesian inertial reference frame. *v_a_* represents the velocity of the aircraft and *ψ* denotes the heading of the UAV. 

In this paper, the angle of attack and the angle of side slip are neglected, then the kinematics equations of each aircraft defined in the inertial frame can be represented as:

(1) $$\begin{array}{l} {\dot{x}}_{i}(t)=v_{a}\cos(\psi_{i}(t))\\ {\dot{y}}_{i}(t)=v_{a}\sin(\psi_{i}(t))\\ \begin{array}{cc} {\dot{\psi }}_{i}(t)=\frac{g\tan\phi_{i}(t)}{v_{g}}, & |\phi_{i}(t)|\leq \phi_{\max} \end{array} \end{array}$$

where *x_i_*(*t*) and *y_i_*(*t*) represent the position of the *i*-th UAV at time instant *t*. *φ_i_*(*t*) denotes the roll angle of the UAV, which is bounded to the range within –*φ*_max_ and *φ*_max_. Assuming that each UAV is equipped with an autopilot so that it is able to follow the given roll angle, let *T*_s_ denote the sampling rate and we apply zero-order hold to the input signal, then the discrete kinematics of the UAV can be found as:

(2) $$\begin{array}{l} {\dot{\psi }}_{i}(k)=\frac{g\tan\phi_{i}(k)}{v_{g}}\\ x_{i}(k+1)=\frac{v_{g}}{{\dot{\psi }}_{i}(k)}\left[\sin({\dot{\psi }}_{i}(k)T_{s}+\psi_{i}(k))-\sin(\psi_{i}(k))\right]+x_{i}(k)\\ y_{i}(k+1)=\frac{v_{g}}{{\dot{\psi }}_{i}(k)}\left[\cos(\psi_{i}(k))-\cos({\dot{\psi }}_{i}(k)T_{s}+\psi_{i}(k))\right]+y_{i}(k)\\ \psi_{i}(k+1)={\dot{\psi }}_{i}(k)T_{s}+\psi_{i}(k) \end{array}$$

If the input roll angle is zero, Equation (2) can be rewritten as:

(3) $$\begin{array}{c} x_{i}\left(k+1\right)=vT_{s}\cos\left({\dot{\psi }}_{i}\left(k\right)\right)+x_{i}\left(k\right)\\ y_{i}\left(k+1\right)=vT_{s}\sin\left({\dot{\psi }}_{i}\left(k\right)\right)+y_{i}\left(k\right)\\ \psi_{i}\left(k+1\right)=\psi_{i}(k) \end{array}$$

### 2.2. Sensor Visible Regions in Complex Environment 

**Figure 2:**
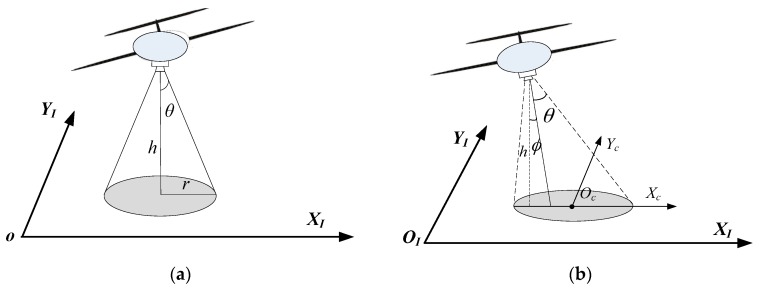
Illustration of the sensor convergence areas with different types of sensors. (**a**) illustrates the visibility regions of gimbaled sensors when the aircraft is turning; (**b**) depicts the visibility regions of body-fixed sensors when the aircraft is turning.

The sensors carried by the UAV can be categorized into to two groups, in terms of the installation conditions, namely gimbaled sensors and body fixed sensors. As shown in Figure 2a, gimbaled installed sensors are capable of pointing to a fixed direction regardless of the attitude of the UAV. The sensor converge area is a cone-shaped region under the aircraft. The pointing orientation of body fixed sensors, on the other hand, are usually affected by the state of the UAV, such as the roll and pitch angles, as shown in Figure 2b. To simplify the problem, in this work UAVs are assumed to be equipped with gimbaled sensors. Hence the sensor convergence on the ground would not be affected by the changes of UAV’s attitude. 

**Figure 3:**
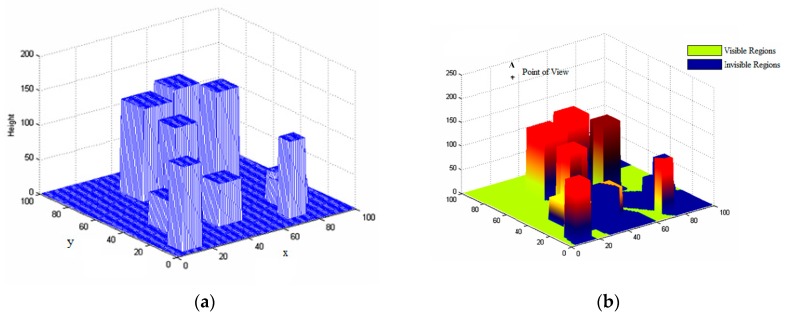
Illustrates of the sensor visible areas in complex environments. (**a**) The simulated urban environment with buildings (rectangular structures); (**b**) shows the visibility regions of a sensor located at the point **A**.

In many applications, the target to be tracked is moving in a complex environment, which may be occluded by terrain features, such as buildings in an urban environment. Figure 3 illustrates a simulated urban environment, where the 3D rectangular structures represent buildings. Due to the occlusion of buildings, not all of the convergence area of the sensor is visible. The actual visible area depends on the height and the field of view of the sensor. As shown in Figure 3, assuming the sensor is located at (*sx*, *sy*, *sz*), the visible regions on the ground can be determined calculated by using the line of slight (LoS) methods. Rather than using conventional geometrical analysis approaches, such as LoS algorithms, to define the visible areas of the sensors, we employ a fast yet robust spatial analysis method developed in [17], which greatly improves the efficiency of this procedure. 

Figure 3b depicts the visible areas looked from the point **A**, the blue regions are invisible areas due to the occlusion of buildings or terrains. Hence, the detectable regions of the sensor can be defined as:

(4) $$R_{vis}(p)=R_{con}(p)\cap R_{in}(p)$$

where *R_con_* is the convergence region of the sensor positioned at *p*, and *R_in_* denotes all of the possible visible areas visible from *p*. Given the field of view angle of the gimbaled sensor *θ*, and the height of the sensor *h*, the convergence region on the ground can be calculated as:

(5) $$\begin{array}{ccc} R_{con}=\pi r^{2} & , & r=h\tan\theta \end{array}$$

### 2.3. The Motion of the Ground Target

The ground object to be tracked is assumed to move within the 2-dimensional plane, and the associated motion can be described by a state vector $x_{t}=\left[x_{t},\;y_{t},\;{\dot{x}}_{t},\;{\dot{y}}_{t},\;{\ddot{x}}_{t},\;{\ddot{y}}_{t}\right]$. Let ***F***() represent the state-transition function of the target dynamic, the motion of the target can be described as follows:

(6) $$x_{t}(k+1)=F[x_{t}(k)]+W(k)$$

where ***W***() denotes the state noise and is usually assumed to be subject to a zero-mean Gaussian distribution, i.e., p(**W**)~N(0, **Q**), **Q** is the covariance matrix. The possible motion of the target can be predicted by filtering approaches [13]. 

## 3. The Proposed Method 

In this paper, we present a solution to the problem of tracking by multiple coordinated UAVs based on the concept of the Receding Horizon Control (RHC) method, which minimizes the uncertainties arising from the application environment and the motion of the target. The associated optimal control sequence estimation problem is then solved by using a novel bio-inspired optimization approach, based on the framework of chemical reaction optimization.

### 3.1. The Receding Horizon Control in Coordinated Tracking

Since the motion of the target is not available as prior knowledge, it requires predicting the trajectory of the target based on previous observation. Receding horizon control (RHC) approach is an advanced model predictive controller allows for determining the optimal solution at current time instant while taking the future states of the target into account. Let *u*[*k*:*k* + *L*] denote the control sequence starts from the time *k*, and *L* is a constant indicating the window size of the RHC sequence. ***X***[*k*] represents the state of the dynamic system, including the positions of the UAV and the target, respectively. The main steps of RHC method for solving the target tracking problem are as follows:

*Step 1*: Determine the optimal control sequence based on the state ***X*** [*k*]:
  (7) $$u^{*}[k:k+L]=\arg\min J(X[k],u[k:k+L])$$
  where u* denotes the optimal control sequence minimizing the cost function *J*. Since the dynamic model of the UAV is obtained from prior knowledge, the trajectory of the UAV can be updated in an iterative fashion based on the input control signal and its current state. Because the motion of the target is unknown, its future positions can only be estimated based on previous observation. The cost function *J* returns the goodness of the tracking based on the relative position between the target and the UAV, which would be discussed in detail in Section 3.2.
*Step 2*: Apply the optimal input sequence u* to the system, and update the states of the system.
*Step 3*: Repeat Steps (1) and (2) for the next time instant.

### 3.2. The Cost of the Tracking Path

#### 3.2.1. Dynamic Constraint of the UAV

In practice, the roll angle of the UAV should be within a reasonable scale when changing its orientation, due to safety considerations. Hence, the input roll angle should satisfy the following constraint:

$$\phi \in \left(-\phi_{\max},\phi_{\max}\right)$$

where *φ*_max_ is a constant representing the maximal roll angle of the UAV during turning process. 

#### 3.2.2. The Target Observation Time

The objective of the coordinate target tracking is to maintain the target located within the detectable region of at least one UAV at any time instant. In addition, it favors the targets located next to the center of the sensor visible area. Let (*rx*, *ry*) denote the relative position of the target with respect to the center of the visible area of the sensor:

(8) $$J_{s}=\sum_{i=1}^{N}\sum_{k=1}^{k+L}sen(X[k])$$

$$Sen=\left\{ \begin{array}{ll} -Ce^{-\frac{rx^{2}+ry^{2}}{2\sigma^{2}}}, & \text{the target is within the visible region of at least one sensor}\\ P, & \text{the target is out of the visible region for all sensors} \end{array}\right.$$

where the function *Sen*() quantifies whether the target can be observed by the sensors of UAVs based on the relative position between the UAVs and the target, This term reaches its minimum if the target is located within the center of the visible area of all of the sensors, and approaches zero if the target is moving to the boundaries of the sensor visible regions. P is a positive constant used to penalize the case where the target is out of the visible region of all of the sensors. 

#### 3.2.3. The Anti-Collision Constraint

In this paper, we assume the UAVs applied to track the target are distributed on the same flight level, thus it is necessary to prevent the UAV from colliding with each other during tracking:

(9) $$J_{AC}=\sum_{i=1}^{N}\sum_{j\in \Omega (i)}erfc\left[k\cdot \left(\left\|x_{i}-x_{j}\right\|-d\right)\right]$$

where *k* is a constant, *d* indicates the desired distance between UAVs, ***x****_i_* and ***x****_j_* represent the positions of *i*-th and *j*-th UAVs in the inertia reference frame, respectively. *erfc* denotes the Gaussian error function which is defined as:

(10) $$erfc(x)=\frac{2}{\sqrt{\pi }}\int_{x}^{\infty }e^{-x^{2}}dx$$

This term would reach its minimum when the positions of the UAVs are evenly distributed around the target. 

#### 3.2.4. The Regulation Term 

In order to ensure the planned path is smooth, the change of the input signal is used as the regulator to penalize a jagged path:

(11) $$J_{reg}=\sum_{i=1}^{N}\sum_{j=k+1}^{k+L}\left(u_{i}(j)-u_{i}(j-1)\right)$$

This term approaches zero when a straight path is generated, and it would be large in magnitude if the resulting path is highly curved. The total cost can be obtained by summing the aforementioned terms together, leading to the following function:

(12) $$J=w_{1}J_{s}+w_{2}J_{AC}+w_{3}J_{reg}$$

where *w*_1_, *w*_2_ and *w*_3_ are constants controlling the influence of each term on the total energy.

### 3.3. Chemical Reaction Optimization-Based Coordinated Tracking 

The Chemical Reaction Optimization (CRO) algorithm, first reported by Lam and Li [16], is a nature-inspired optimization algorithm based on the principle of chemical reactions. It mimics the process of chemical reactions occuring in a closed container. CRO is a type of population-based algorithm where the basic individuals are molecules. Each molecule is described by some attributes, including the molecular position, i.e., a possible solution of the optimization problem, the potential energy (PE) and the kinetic energy (KE). The molecule structure represents a possible solution of the optimization problem, the potential energy (PE) is associated with the cost of the resulting solution based on the objective function. In general, a small value of the potential energy may indicate a better solution of the optimization problem. Kinetic energy (KE) defines the tolerance of the system for accepting a worse solution than the existing one. In addition to molecules, the container (buffer), where the chemical reaction takes place, is another key component of the CRO algorithm. It is able to supply or absorb the energy resulting during the process of the molecular reactions. Different from many existing population-based optimization algorithms, the total number of molecules may not be the same in each iteration of the optimization process, but the entire amount of energy (the energies of molecules and the energy of the buffer) of the system stays constant throughout the reaction process. 

#### 3.3.1. Coding Scheme

**Figure 4:**
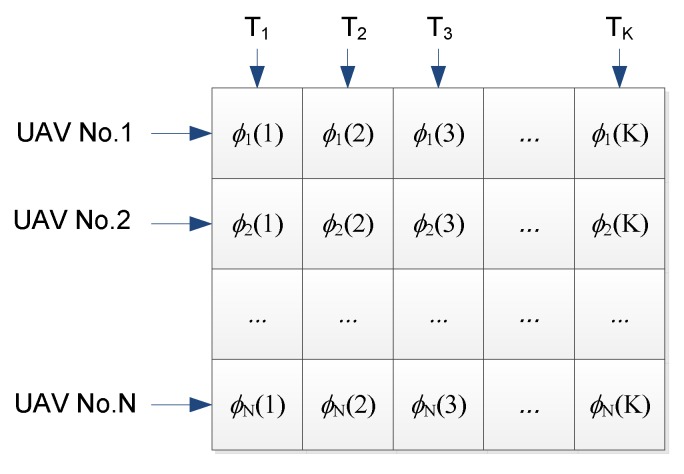
The coding scheme of the CRO algorithm.

As shown in Figure 4, the molecular structure is defined as a sequence of bounded input signals, i.e., the desired roll angle to the autopilot in the time from t_*k*_ to *t_k_*_+1_ for each UAV. Thus, the dimensions of each molecule would be *N* × *L*, where *N* represents the total number of UAVs employed for coordinated tracking, and *L* is the number of steps in the predicted ahead current state.

#### 3.3.2. On-Wall Ineffective Collision

In this elementary process, the new path is determined by slightly deforming the current path, which can be considered as a local search around the current solution. Let *KE*_ω_ and *PE*_ω_ represent the kinetic and potential energy of a molecule before it hitting the wall of the container, respectively. The potential energy can be calculated based on Equation (12), and the kinetic energy for each molecule is initialized randomly. Due to the collisions, a certain amount of the energy may transfer to the wall of the container (i.e., the buffer), and thus the potential *PE*_ω*′*_ and kinetic energy *KE*_ω*′*_ of such molecule at the new statue can be computed as:

(13) $$\begin{array}{cc} KE_{\omega^{\prime }}=(PE_{\omega }+KE_{\omega }-PE_{\omega^{\prime }})\times \alpha , & \mathrm{if}\;(PE_{\omega }+KE_{\omega }-PE_{\omega^{\prime }})\geq 0 \end{array}$$

Let *KElossRate* (*KElossRate* ϵ [0, 1]) be a constant defining the percentage of energy that lost before and after the molecule hits the wall, *α* is a random number evenly distributed from *KElossRate* to 1. Based on the law of conversion of energy, the energy absorbed by the buffer can be expressed as:

(14) $$KE_{\omega^{\prime }}=(PE_{\omega }+KE_{\omega }-PE_{\omega^{\prime }})\times (1-\alpha )$$

If the kinetic energy of the molecule before wall collision is large enough, the after collision potential energy may larger than *PE*_ω_, leading to a worse solution when compared with the existing one. It allows the local search algorithm the ability of ‘hill-climbing’, which increases the probability of avoiding local optimal solutions.

#### 3.3.3. Decomposition

Unlike the process of the ‘on-wall ineffective collision’, the molecule breaks into parts after it hits the wall of the container (*ω*→*ω*_1_*ʹ* + *ω*_2_*ʹ*). For simplicity we only consider two parts. Because more solutions are created through the decomposition process, the sum of KE and PE energies of the original molecule may be less than the sum of potential energies of the resulting molecules:

(15) $$PE_{\omega }+KE_{\omega }<PE_{{\omega^{\prime }}_{1}}+PE_{{\omega^{\prime }}_{2}}$$

In this case, such decomposition is not valid since it disobeys the law of energy conservation. In order to increase the possibility of a correct decomposition process, the buffer may supply a certain amount of the energy to the decomposition process so that the following condition can be satisfied:

(16) $$PE_{\omega }+KE_{\omega }+\delta_{1}\times \delta_{2}\times buffer\geq PE_{{\omega^{\prime }}_{1}}+PE_{{\omega^{\prime }}_{2}}$$

where *δ*_1_ and *δ*_2_ are two independently random variables uniformly distributed in the range of [0, 1]. The remaining energy is translated as the kinetic energies for two new molecules as:

(17) $$\begin{array}{l} PE_{{\omega^{\prime }}_{1}}=(PE_{\omega }+KE_{\omega }+\delta_{1}\times \delta_{2}\times buffer-PE_{{\omega^{\prime }}_{1}}-PE_{{\omega^{\prime }}_{2}})\times \delta_{3}\\ PE_{{\omega^{\prime }}_{2}}=(PE_{\omega }+KE_{\omega }+\delta_{1}\times \delta_{2}\times buffer-PE_{{\omega^{\prime }}_{1}}-PE_{{\omega^{\prime }}_{2}})\times (1-\delta_{3}) \end{array}$$

where *δ_3_* is another independent random variable range from 0 to 1. The decomposition process provides the molecule the ability of exploring more solution regions with respect to the initial position *ω.*

#### 3.3.4. Inter-Molecular Ineffective Collision

The reaction process is very similar to its single molecule reaction counterpart, where the collision takes place between molecules. The number of the molecules is assumed to be unchanged before and after the collision, and the molecular energies of the associated reaction process should satisfy the condition:

(18) $$PE_{\omega_{1}}+KE_{\omega_{1}}+PE_{\omega_{2}}+KE_{\omega_{2}}\geq PE_{{\omega^{\prime }}_{1}}+PE_{{\omega^{\prime }}_{2}}$$

The kinetic energies of the new molecules are:

(19) $$\begin{array}{l} KE_{{\omega^{\prime }}_{1}}=E_{\mathrm{inter}}\times \delta_{4}\\ KE_{{\omega^{\prime }}_{2}}=E_{\mathrm{inter}}\times (1-\delta_{4})\\ E_{\mathrm{inter}}=PE_{\omega_{1}}+KE_{\omega_{1}}+PE_{\omega_{2}}+KE_{\omega_{2}}-PE_{{\omega^{\prime }}_{1}}+PE_{{\omega^{\prime }}_{2}} \end{array}$$

The inter-molecule collision allows a median range local search, thus increasing the probability of detecting a better solution in a local area. 

#### 3.3.5. Synthesis

Synthesis is the opposite reaction process of decomposition, where two (multiple) molecules merge into a single molecule. As the new molecule is produced from multiple molecules, it is more likely to meet the following requirement for such a process:

(20) $$PE_{\omega_{1}}+KE_{\omega_{1}}+PE_{\omega_{2}}+KE_{\omega_{2}}\geq PE_{\omega^{\prime }}$$

The remaining energy of the new molecule is:

(21) $$KE_{\omega^{\prime }}=PE_{\omega_{1}}+KE_{\omega_{1}}+PE_{\omega_{2}}+KE_{\omega_{2}}-PE_{\omega^{\prime }}$$

In this reaction process, the new molecule is a combination of multiple old molecules, thus the newly produced molecule may exhibit a large deviation from the original ones in the solution space. 

**Figure 5:**
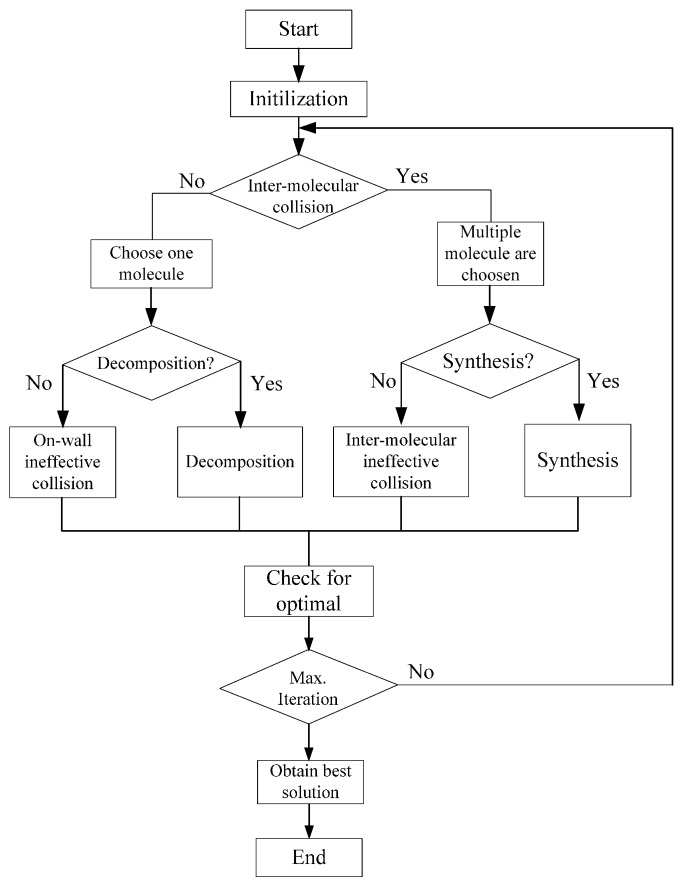
Schematic diagram that illustrates the CRO optimization framework.

The workflow of the CRO based optimization framework is shown in Figure 5.

## 4. Simulation Results and Analysis

This section presents comparative simulation results to demonstrate the efficiency and accuracy of the proposed technique in the coordinated UAV tracking problem. The simulations were conducted using 3D synthetic environmental data, which allows for generating a variety of virtual environments with different resolutions and urban features. The simulations were carried out in MATLAB (R2010b, MathWorks, Natick, MA, USA) on a standard specification PC (Dell Precision T3500, DELL, Round Rock, TX, USA, Inter(R) Xeon(R) CPU operating at 2.67 GHz). 

The dimensions of the virtual environment are defined within the range of 1.5 km × 1.5 km, and 30 buildings with different dimensions are randomly generated. Three UAVs are employed to track one moving target, and the motion of the UAVs and target are described using the dynamic model discussed in Section 2. The flight speed *v*_g_ for each UAV is set to be 30 m/s, the flight level is 200 m, the maximum roll angle *φ*_max_ is set to 45° and the minimal distance between each UAV is 50 m. The trajectory of the target is randomly generated in the flexible region on the ground, with a constant speed at 15 m/s. The RHC window size *L* is set as 5 s, and the total simulation time is 100 s, and the sampling time is 1 s. The parameter settings of the CRO algorithm are shown in Table 1. 

**Figure 6:**
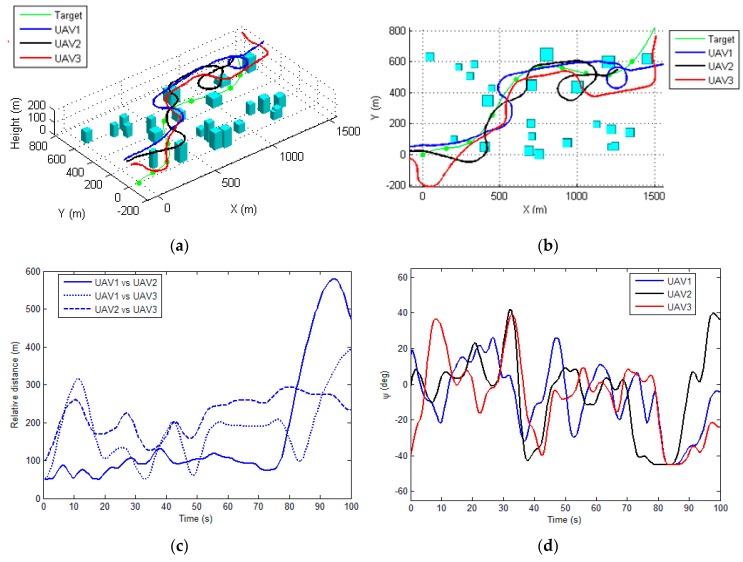

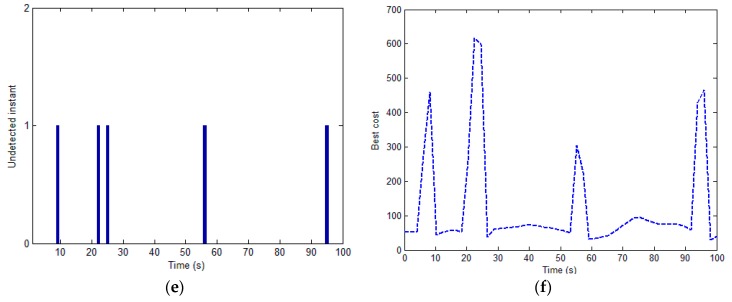
The performance of the proposed method for scenario 1. (**a**) 3D view of the planned path for each UAV; (**b**) Top view of the resulting paths; (**c**) The relative distance among each UAV during tracking; (**d**) The roll-command for each UAV (**e**) The undetected time-instant; (**f**) The total cost of the tracking process.

**Figure 7:**
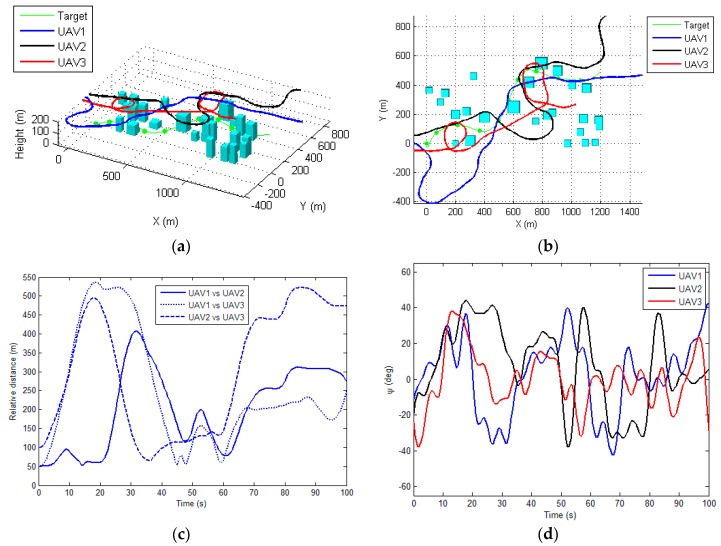

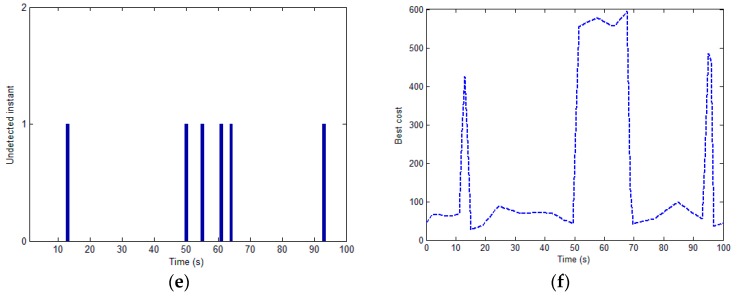
The performance of the proposed method for scenario 2. (**a**) 3D view of the planned path for each UAV; (**b**) Top view of the resulting paths; (**c**) The relative distance among each UAV during tracking; (**d**) The roll-command for each UAV (**e**) The undetected time-instant; (**f**) The total cost of the tracking process.

Figure 6 and Figure 7 illustrate the performance of the proposed method for tracking the moving target in two scenarios. The total visibility time for these two scenarios are 95 s and 94 s (as shown in Figure 6e and Figure 7e, respectively), which approach the total flight time (100 s). This indicates that the proposed method is able to generate an optimal tracking path for each UAV. The costs of the proposed method at each sampling time are depicted in Figure 6f and Figure 7f, respectively. The cost grows rapidly when the target is out of the visibility region for all of the sensors, and for the rest the time the costs are almost constant, implying that the proposed algorithm can determine the optimal path within the sampling time (1 s). As shown in Figure 6d and Figure 7d, the control input for each UAVs, i.e., the roll angle, was changing frequently during the tracking process, since the UAVs are required to maneuver to track the moving target while avoid the occlusions of the buildings. The control inputs determined by the proposed method are generally smooth and changing slowly, which can be tracked by the autopilot of the UAV. 

Figure 6c and Figure 7c illustrate the relative distances between each UAV during the tracking process for two scenarios, respectively. It can be seen that the generated path by the proposed method always satisfies the safety constraint (i.e., the minimal distance between each UAV). In order to demonstrate the efficiency of the proposed CRO based cooperative tracking path planning algorithm, this section firstly compares the proposed approach with commonly used evolutionary algorithms based optimizer, i.e., the genetic algorithm (GA) [13] and particle swarm optimization (PSO) [14], for determination of the tracking paths. 

**Figure 8:**
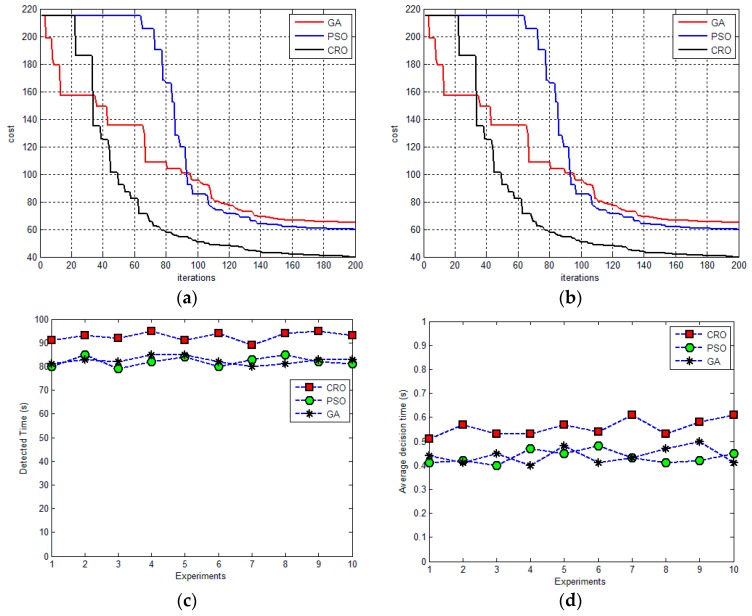
The performances of using different evolutionary optimizers for determination of the tracking path for scenario 1. (**a**) and (**b**) illustrate the convergence of different methods in selected decision time, respectively; (**c**) shows the visibility time of the ground time for the proposed method, PSO and GA based algorithms, respectively; (**d**) is the average execution time of the aforementioned methods.

**Figure 9:**
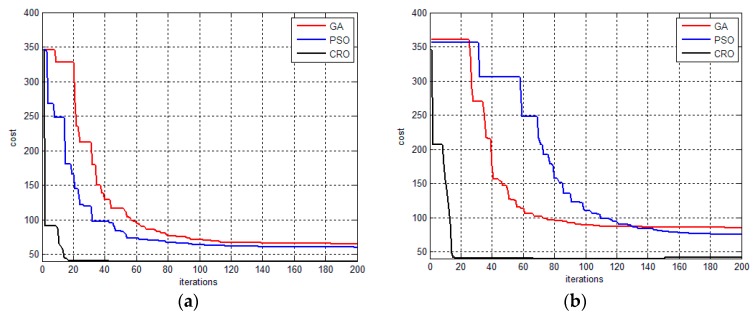

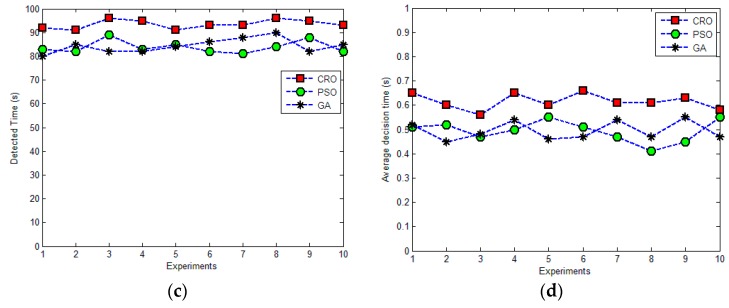
The performances of using different evolutionary optimizers for determination of the tracking path for scenario 2. (**a**) and (**b**) illustrate the convergence of different methods in selected decision time, respectively; (**c**) shows the visibility time of the ground time for the proposed method, PSO and GA based algorithms, respectively; (**d**) is the average execution time of the aforementioned methods.

Figure 8 and Figure 9 illustrate the performances of the proposed method, GA-based method and PSO- based algorithm for the tracking task, respectively. Figure 8a,b and Figure 9a,b illustrate the convergence of the proposed method and the other evolutionary-based optimizers from randomly selected sampling times for two scenarios. It can been seen that the proposed approach outperforms to GA- and PSO-based optimizer in terms of convergence speed and optimality (in terms of the cost value). Figure 8c and Figure 9c depict the visibility time of the ground target of the aforementioned algorithms obtained from 10 repeat simulations. The overall performance of the proposed method is better than GA- and PSO-based planners in terms of the visibility time. This is because the CRO is a variable population-based metaheuristic approach, which allows for generating more solutions near the optimality and thus increasing the convergence speed. Figure 8d and Figure 9d are the average execution times for each decision point (i.e., the sampling point), where the proposed method requires more time than the GA- and PSO-based planners. However, this execution time (around 0.55 s for the proposed method) is acceptable when compared the sampling time (1 s). 

**Figure 10:**
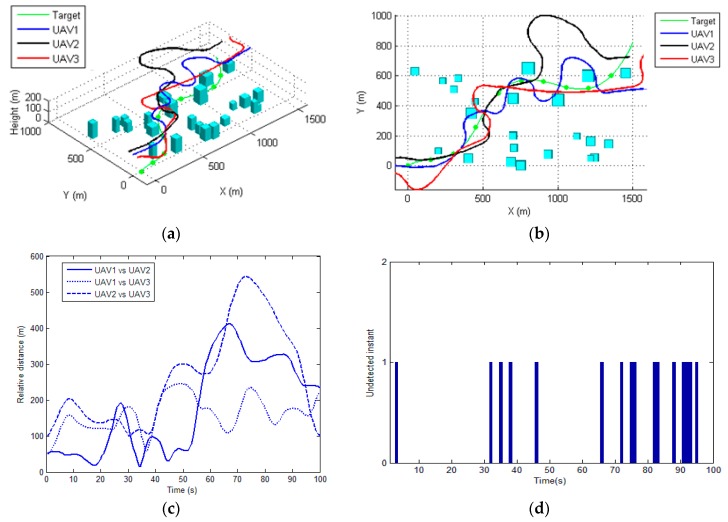
The results obtained by using LGVF based approach for scenario 1. (**a**) 3D view of the planned path for each UAV; (**b**) Top view of the resulting paths; (**c**) The relative distance between each UAV during tracking; (**d**) The undetected time-instant.

**Figure 11:**
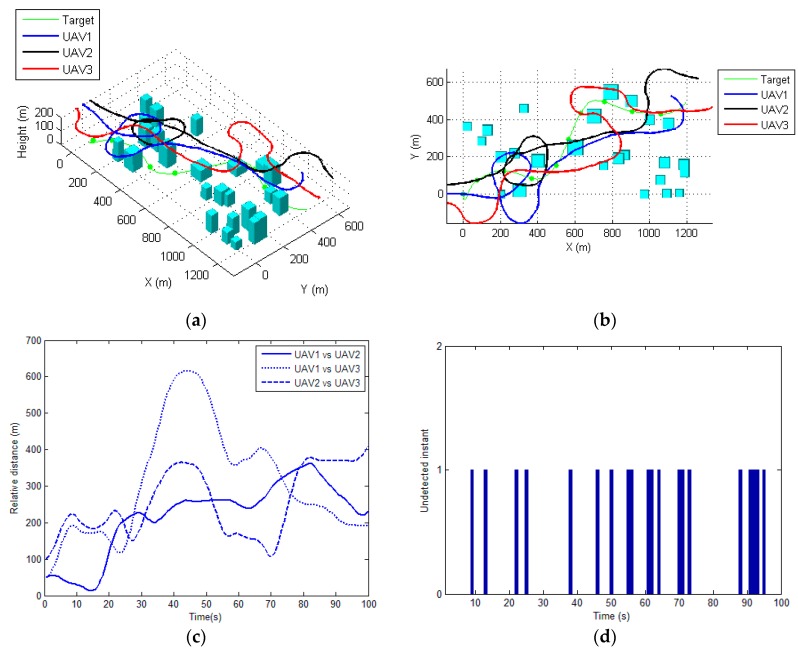
The results obtained by using LGVF based approach for scenario 2. (**a**) 3D view of the planned path for each UAV; (**b**) Top view of the resulting paths; (**c**) The relative distance between each UAV during tracking; (**d**) The undetected time-instant.

Next, the proposed method is compared with a recently developed Lyapunov guidance vector field (LGVF)-based approach for planning the cooperative tracking path [18]. Figure 10 and Figure 11 illustrate the results of using the LGVF-based approach for two scenarios. It can be observed that the proposed method achieves better solution to the cooperative path planning problem in terms of the visibility time. The minimal distances between UAVs are not always satisfactory (i.e., less than the safety distance, i.e., 50 m), as shown in Figure 10c and Figure 11c. The statistics of the proposed method and the LGVF-based approach obtained from 10 repeat simulation tests are shown in Table 2 and Table 3, respectively. The mean, maximum and minimum visibility times of the proposed method are better than those of the LGVF-based planner. This is because it is difficult for the LGVF-based optimizer to incorporate nonlinear and non-convex constraints, such as the visibility of sensors and anti-collision constraints in the tracking optimization problem, and thus cannot always reach the optimal solution.

## 5. Conclusions

Tracking moving targets using UAVs is a challenging task due to the uncertainties arising from the motion of the target and the environmental features. This paper presents a novel method, coupling the concept of model predictive control into the framework of chemical reaction optimization, to solve the coordinated tracking path planning problem in complex urban environments. The sensor visible regions under urban environment, flight dynamics and anti-collision constraints are considered. Particularly, the LoS occlusion caused by dense buildings is discussed, and the FOV for a gimbaled sensor is also analyzed. A series of comparative simulations have shown that the proposed method outperforms other metaheuristic and mathematical methods in terms of tracking ability and safety, while achieving similar computational efficiency. The simulation results imply that the proposed method is capable of improving the performance of target tracking in urban environments, with more visible time, higher security and stronger robustness compared to other methods. 

## Figures and Tables

**Table 1:** Parameter settings of CRO algorithm.

KELossRate	0.2	InitialKE	200
MoleColl	0.3	PopSize	100
buffer	0	α	500
Β	10	c_1_	0.15
c_2_	2.5		

**Table 2:** Statistics results of the tracking performances for scenario 1.

Performance	Proposed Method	LGVF-Based Algorithm
Mean visibility time (s)	95	82
Maximum visibility time (s)	96	79
Minimum visibility time (s)	91	84
Variation of visibility time (s)	4	3
Range of relative distances (m)	[51, 588]	[11, 548]

**Table 3:** Statistics results of the tracking performances for scenario 2.

Performance	Proposed Method	LGVF-Based Algorithm
Mean visibility time (s)	94	83
Maximum visibility time (s)	97	81
Minimum visibility time (s)	91	89
Variation of visibility time (s)	4	7
Range of relative distances (m)	[54, 608]	[15, 611]
